# Child educational progress in Born in Bradford pregnancies affected by gestational diabetes and also exposed to maternal common mental disorders

**DOI:** 10.1038/s41598-023-44619-0

**Published:** 2023-10-21

**Authors:** Claire A. Wilson, Gillian Santorelli, Louise M. Howard, Khalida Ismail, Rebecca M. Reynolds, Emily Simonoff

**Affiliations:** 1 Institute of Psychiatry, Psychology and Neuroscience, King’s College London, De Crespigny Park, PO31, London, SE5 8AF UK; 2 South London and Maudsley NHS Foundation Trust, London, UK; 3 Born in Bradford, Bradford Teaching Hospitals NHS Foundation Trust, Bradford, UK; 4 Centre for Cardiovascular Science, University of Edinburgh, Edinburgh, UK

**Keywords:** Health care, Risk factors

## Abstract

Gestational diabetes and the maternal mental disorders of anxiety and depression have been implicated in adverse offspring neuro-behavioural outcomes but these exposures have only been studied in isolation. 1051 children whose mothers were diagnosed with gestational diabetes in UK’s Born in Bradford cohort had linkage to maternal primary care records, providing diagnostic and treatment codes for depression and anxiety. Education record linkage provided results of the Early Years Foundation Stage Profile from the first year of school, aged five. Risk of not attaining a ‘Good level of development’ was analysed using multivariable Poisson regression within a generalised estimating equation framework. Multiple imputation was implemented for missing data. There was limited evidence of increased risk of failure to attain a ‘good level of development’ in those additionally exposed to maternal mental disorders (adjusted RR 1.21; 95% CI 0.94, 1.55). However, there was more evidence in children of Pakistani maternal ethnicity (adjusted RR 1.36; 95% CI 1.04, 1.77) than White British; this may have been driven by English not being the primary language spoken in the home. Therefore there may be groups with GDM in whom it is particularly important to optimise both maternal physical and mental health to improve child outcomes.

## Introduction

It is now widely acknowledged that the in-utero environment to which the developing fetus is exposed influences future health outcomes; this is known as ‘developmental programming’^1^. Two prevalent adversities to affect the developing fetus are gestational diabetes (GDM) and the common mental disorders (CMD) of anxiety and depression.

GDM is glucose intolerance that arises for the first time during pregnancy and usually resolves following delivery. It affects between five and 10% of pregnant women^2^. CMD is also common, likely affecting at least 10% of pregnant women^3^. Moreover, there is accumulating evidence that these two conditions may frequently co-occur^4^.

There is some evidence that GDM and maternal antenatal CMD are associated with poorer neuro-behavioural outcomes in offspring. These include a range of adverse cognitive, emotional and behavioural sequelae common to both conditions, such as delays in early cognitive, language and motor development and an increased risk of neurodevelopmental and psychiatric disorders^5–8^. To date the pathways impacting offspring development stemming from the two exposures have only been studied in isolation (GDM leading to adverse outcomes or antenatal CMD leading to adverse outcomes). These developmental pathways are clearly complex, with a range of potential moderating and mediating factors along the developmental trajectory^1,6^. The impact on offspring development of exposure to these combined in-utero exposures of GDM and CMD has not yet been studied.

Despite this, the developmental outcomes of children born to women who have been affected by both GDM and antenatal CMD is important to investigate, not only because of the high levels of comorbidity between GDM and CMD, but also because there is some evidence that those experiencing both diabetes and mental disorder may have poorer glycaemic control than those without mental disorder^9^, and there is also evidence to support an inverse dose response relation between level of maternal hyperglycaemia and offspring outcomes such as IQ and motor development^10–13^.

Thus the aim of this study was to compare educational progress at age five in children exposed to GDM alone compared to those exposed to both GDM and CMD during pregnancy. It was hypothesised that children born to women with both GDM and CMD would have an increased risk of adverse outcomes compared to children born to women with GDM without CMD. It was also hypothesised that this risk may differ between ethnic groups, as there is evidence that the impact of maternal CMD on child development may vary according to ethnicity^14^.

## Methods

Methods were performed in accordance with ‘strengthening the reporting of observational studies in epidemiology’ (STROBE) guidelines^15^.

### Sample: children whose mothers had GDM

Born in Bradford (BiB) is a prospective longitudinal cohort of 12,450 women with 13,758 pregnancies in Bradford, UK. Pregnant women were recruited to the BiB cohort between 2007 and 2010^16^ when attending a routine appointment for a two hour 75 g oral glucose tolerance test (OGTT), as currently recommended by the UK’s National Institute for Health and Care Excellence (NICE) as the gold standard for diagnosing GDM^17^. The test was offered to all women in Bradford, usually between 26 and 28 weeks gestation. Consent was obtained for record linkage to maternity and primary care records via SystmOne: a clinical computer system used by almost all general practices in Bradford and which provides primary care data on diagnoses and prescriptions.

**Figure 1:**
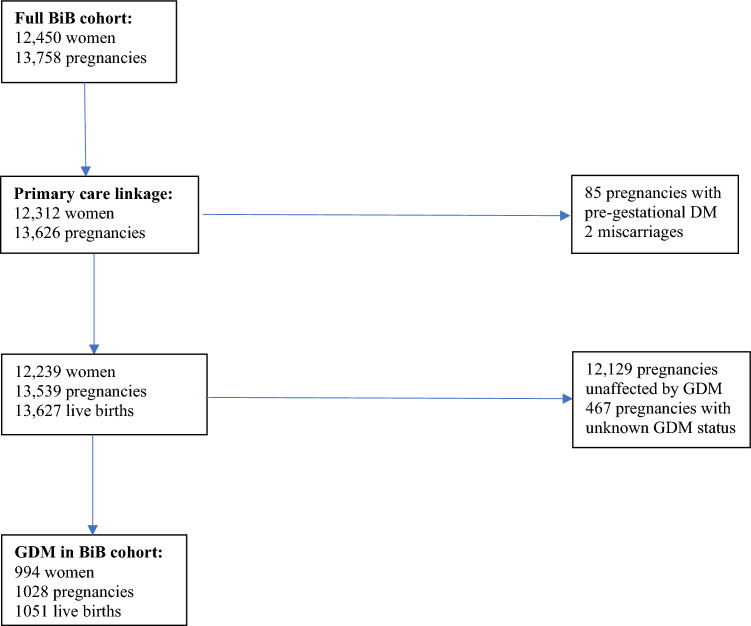
How the sample was obtained.

GDM was diagnosed according to modified World Health Organisation (WHO) criteria (either fasting glucose ≥ 6·1 mmol/l or 2 h post-load glucose ≥ 7·8 mmol/l)^18^. The sample used for analysis was live births from women who had both GDM and linkage to primary care records (n = 994 women with 1051 live births; Fig. 1).

### Maternal exposure: antenatal (during pregnancy) CMD

As an indicator of CMD, Read codes (Clinical Terms Version 3) for diagnosis and/or treatment of CMD were extracted from GP electronic health records alongside medication prescriptions as per previously published methods (supplementary material S1)^19^. The binary variable of antenatal CMD was defined as any indicator of CMD appearing in the primary care notes between the date of conception (estimated from date of last menstrual period and/or booking scan) and the date of delivery.

### Offspring outcome: failure to meet a good level of development on the early years foundation stage profile

There is also linkage within the BiB cohort to the child’s education records, providing the results of national assessments. The earliest national collection of data on educational progress from school-aged children in England is the Early Years Foundation Stage Profile (EYFSP), completed at the end of reception year (the first year of primary school) when children are aged five. Teachers are asked to rate children across a number of different aspects of learning, based on their knowledge and accumulating observations of the child throughout the school year.

The most widely used indicator of child development at this age is a ‘good level of development’ (GLD) on the EYFSP, which is being rated by the teacher as meeting expectations across five of the areas covered by the EYFSP: (1) communication and language, (2) literacy, (3) mathematics, (4) physical development, (5) personal, social and emotional development^20^. This binary variable of failure to attain GLD (yes or no) was used as the primary outcome.

### Covariates

There were a number of variables identified as potential confounders^21^. These were maternal age at the time of the OGTT^22,23^, tobacco smoking during pregnancy^24,25^, singleton versus multiple pregnancy^26,27^ and other obstetric complications (pre-eclampsia and/or gestational hypertension)^28,29^. CMD prior to pregnancy (‘preconception’ CMD) was also measured using Read codes and medication prescriptions in the primary care record from the woman’s birth to the date of conception of the woman’s first ever pregnancy (supplementary material S1). Also included was maternal socioeconomic status (SES)^23,30^; maternal education was used a proxy, as the high levels of deprivation in BiB result in a highly skewed distribution of participants across deprivation categories.

It was also hypothesised that the continuous variable of fasting glucose on the OGTT and the binary variable of GDM treated with or without insulin may be effect modifiers. Other potential moderators considered were sex of the child^31^, season of birth of the child^32^ and maternal pre-pregnancy body mass index (BMI)^33^.

### Statistical analysis

Data were analysed using Stata version 15^34^. Multivariable robust Poisson regression was employed in the sample of children exposed to GDM to examine the association between the additional exposure of maternal CMD and failure to attain GLD, within a generalised estimating equation framework using the robust sandwich estimator for standard errors to account for clustering from those women with more than one child in the sample (either due to twin or triplet pregnancies or multiple pregnancies occurring during the data collection period). Two analyses were performed: unadjusted and adjusted for the confounders described above. Interaction terms were also included in the models for those moderators reported above.

Multiple imputation by chained equations was implemented to handle missing data^35^. 40 imputations were used to reflect the proportion of participants with any missing data^36^. All variables used in the primary analysis were included in the imputation model and an additional variable of being in receipt of child benefit payments or not was used as an auxiliary variable, as it was associated with being an incomplete case. Estimates were obtained by pooling results using Rubin’s rules^37^. Complete case analyses were also conducted.

### Subgroup analyses

The above analyses were stratified into two groups of children with known Pakistani and White British maternal ethnicity. As these were the most prevalent ethnicities, further categories of ethnicity such as ‘Other’ would have been too heterogeneous.

### Sensitivity analyses

The following analyses were performed in addition to the primary analysis:

1. Analysis of the stratified samples after removal of children with English as an additional language (EAL) status in their education records. EAL is defined as English not being the primary language spoken in the family home^38^.
2. In the stratified samples, risk for failing to attain GLD in each of the five EYFSP sub-domains described above.

### Ethics

Ethical approval for data collection in BiB was granted by Bradford Research Ethics Committee (Ref 07/H1302/112). Informed consent was obtained from all participants.

## Results

The demographics of BiB are broadly representative of Bradford as a whole^16^. Table 1 displays the characteristics of the sample used for analysis and the proportion of missing data in each variable, alongside imputed values for those variables that were imputed. Compared to the wider BiB cohort, those women affected by GDM were older, had lower educational attainment and were more likely to be of Pakistani ethnicity^16^. 10% of children were exposed to antenatal CMD. 39% of children with available data failed to meet a good level of development (GLD) on the EYFSP.

**Table 1:** Characteristics of the sample (N = 1051 children).

	n	%	Imputed%
Maternal ethnicity			
Pakistani	553	52.6	N/A^a^
White British	212	20.2	N/A^a^
Other	136	12.9	N/A^a^
Missing	150	14.3	
Maternal age (years)			
Mean (SD)	30.4	(5.4)	30.4 (5.5)
Missing	147	14	
Maternal education			
Less than 5 GCSE equivalents	231	22	25.9
5 GCSE equivalents	251	23.9	28
A level equivalents	99	9.4	11.1
Higher than A level	251	23.9	27.6
Other	68	6.5	7.5
Missing	151	14.4	
Multiple pregnancy			
Singleton	1006	95.7	N/A^a^
Multiple pregnancy (twins or triplets)	45	4.3	N/A^a^
Maternal tobacco smoking in pregnancy			
Yes	75	7.1	8.5
No	828	78.8	91.5
Missing	148	14.1	
Obstetric complication (pre-eclampsia and/or gestational hypertension)			
Yes	62	5.9	6.5
No	912	86.8	93.5
Missing	77	7.3	
Maternal indicator of preconception CMD			
Yes	139	13.2	14.2
No	824	78.4	85.8
Missing	88	8.4	
Maternal indicator of antenatal CMD			
Yes	107	10.2	N/A^a^
No	944	89.8	N/A^a^
Failure to meet good level of development (GLD) on EYFSP			
Yes	326	31	39.5
No	503	47.9	60.5
Missing	222	21.1	

^a^Data not imputed.

In the sample of 1051 children exposed to GDM, Table 2 displays the unadjusted and adjusted RRs for failure to attain GLD in children additionally exposed to antenatal CMD versus those unexposed to antenatal CMD using imputed data. There was little evidence on unadjusted or adjusted analyses of an effect of antenatal CMD on risk for not meeting GLD in children whose mothers had GDM (adjusted RR 1.21, 95% CI 0.94, 1.55). Results of complete case analyses mirrored those of the imputed results; see supplementary material (S2). There was no evidence for an interaction between antenatal CMD and fasting glucose (p = 0.166) or between antenatal CMD and insulin-treated GDM (p = 0.714). There was also no evidence of effect modification by sex of child (p = 0.351 for the interaction term of antenatal CMD and female sex, comprising 48% of the sample), pre-pregnancy BMI (p = 0.894) or birth of the child in the summer (p = 0.309).

**Table 2:** Associations between antenatal CMD and EYFSP results in children exposed to GDM using imputed data (N = 1051 children).

	Failure to attain good level of development (GLD) on EYFSP			
	%	RR	(95% CI)	p
Unadjusted				
Maternal indicator of antenatal CMD reference category = no indicator	38.6			
Antenatal CMD indicator	46.7	1.19	(0.93, 1.53)	0.170
Adjusted^a^				
Maternal indicator of antenatal CMD reference category = no indicator	38.6			
Antenatal CMD indicator	46.7	1.21	(0.94, 1.55)	0.131

Models using imputed data in a Poisson regression within a generalised estimating equation framework with robust standard errors.

^a^Adjusted for maternal age, maternal education, multiple pregnancy, tobacco smoking in pregnancy, obstetric complications and maternal preconception CMD.

### Subgroup analyses

42% of children from Pakistani maternal ethnicity failed to attain GLD versus 35% of those from White British mothers. Table 3 displays the results of the primary analysis stratified by maternal ethnicity. The direction of effect was observed to differ between children born to women of Pakistani versus White British ethnicity; there was evidence of an increased risk of failure to meet GLD following exposure to antenatal CMD and GDM observed only in the Pakistani group, which was only slightly attenuated on adjustment for confounders. On examining the influence of each confounder in turn, this attenuation appeared to be driven by maternal education.

**Table 3:** Associations between antenatal CMD and EYFSP results in children exposed to GDM using imputed data and stratified by maternal ethnicity.

	Pakistani (N = 553 children)				White British (N = 212 children)			
	Failure to attain good level of development (GLD) on EYFSP				Failure to attain good level of development (GLD) on EYFSP			
	%	RR	(95% CI)	p	%	RR	(95% CI)	p
Unadjusted								
Maternal indicator of antenatal CMD reference category = no indicator	39.7				35.4			
Antenatal CMD indicator	55.5	1.39	(1.06, 1.81)	0.016	29.4	0.76	(0.32, 1.81)	0.532
Adjusted^a^								
Maternal indicator of antenatal CMD reference category = no indicator	39.7				35.4			
Antenatal CMD indicator	55.5	1.36	(1.04, 1.77)	0.023	29.4	0.79	(0.34, 1.86)	0.591

Models using imputed data in a Poisson regression within a generalised estimating equation framework with robust standard errors.

^a^Adjusted for maternal age, maternal education, multiple pregnancy, tobacco smoking in pregnancy, obstetric complications and maternal preconception CMD.

### Sensitivity analyses

72.7% of the GDM affected children of Pakistani maternal origin were recorded as EAL (English as an additional language) in their education records versus 2.3% of those of White British maternal ethnicity. Removal from the GDM exposed sample of children with known EAL resulted in a reduction in risk for failure to attain GLD following antenatal CMD exposure in the Pakistani subgroup (N = 201 children; adjusted RR 1.08, 95% CI 0.57, 2.06) but with widening of the CI as a result of the smaller sample size. In the White British subgroup, fewer children with EAL resulted in little change to the sample and associated effect estimate (N = 208 children; adjusted RR 0.85, 95% CI 0.37, 1.94).

The risk for failing to attain GLD stratified by ethnicity across the seven EYFSP sub-domains are presented in full in supplementary material (S3). The direction of effect of risk following exposure to antenatal CMD in children affected by GDM was similar between ethnic groups for the individual domains of the EYFSP as for the global GLD rating used in the primary analyses. However, the ethnic differences may be more marked in the domain of literacy; there was some evidence for an increased risk for failure to attain GLD following exposure to antenatal CMD in the Pakistani subgroup exposed to GDM on unadjusted analyses, which was attenuated on adjustment for confounders.

## Discussion

### Main findings

In this sample there was limited evidence that children born to women with GDM and antenatal CMD are at an increased risk of failing to meet GLD (a good level of development) compared to children born to women with GDM without CMD. There was some evidence for this in the Pakistani subgroup, although this was not observed when children with EAL (English as an additional language) were removed from the analysis and risk was most elevated in the literacy subdomain. Thus there was some evidence of a combined burden of exposure to the early adversities of GDM and antenatal CMD in groups of children possibly already vulnerable to poorer educational attainment.

### Findings in context

There have been relatively more studies on the offspring neuro-behavioural sequalae of antenatal CMD exposure than GDM and when positive associations are found in these studies, the effect sizes are often relatively small. There are also several moderators that were unmeasured in this study, such as familial social support and quality of parenting^6^, which may help to reverse adverse developmental trajectories from becoming established.

In the GDM literature, indirect correlations have been observed between level of glycaemic control and both IQ scores^39^ and the degree of fine and gross motor deficiency^12,13^ in school-age children, with the impact of oxidative stress on the developing brain implicated in the pathophysiology. However, in this study, neither fasting glucose nor insulin use: as measures of the degree of hyperglycaemia exposure, were significant effect modifiers.

Regarding ethnicity, there are documented differences in educational attainment in the UK, with some ethnic minority groups, including Pakistani, showing poorer outcomes^40^. Indeed language spoken at home appears to be a major driver of attainment^40^. Yet ethnicity is rarely mentioned in reviews of the literature on the impact of GDM or CMD on child outcomes^5,41^. Boys also appear to be relatively more disadvantaged by their non-White ethnicity than girls. However, sex of child did not seem to have a significant impact on risk of failing to meet GLD in this analysis.

The EYFSP is one of the most widely used measures of child development in England at age five and has been widely evaluated^42^. In BiB, poorer EYFSP attainment is associated with autism diagnoses; autism is also associated with exposure to GDM and antenatal CMD^43^. The risks of failing to attain GLD in this study are slightly lower than England-wide figures from the same period, although ethnic differences are also seen (58% of Pakistani children failed to meet GLD versus 43% of White British)^38^. One of the cognitive domains which seems to be particularly vulnerable to the effects of both ethnicity and antenatal CMD is that of language^40,41^, consistent with the ethnic differences in literacy observed in this study. Perhaps it is surprising that ethnic differences were not also seen in communication and language.

### Strengths and limitations

This is the first study to examine the neuro-behavioural outcomes of children exposed to concurrent GDM and CMD. The use of such a diverse sample facilitated exploration of a broad range of variables and has highlighted the importance of considering the wider, social determinants of child development, including ethnicity albeit it was within a smaller sample of GDM affected pregnancies in the wider BiB cohort.

Nonetheless, clearly there may be limitations in the generalisability of this predominantly bi-ethnic (Pakistani and White British) cohort, with such high levels of socioeconomic deprivation. Another unique characteristic of BiB are the high levels of consanguinity. Indeed infant mortality in Bradford is among the highest in the UK and the levels of congenital anomalies are also high^16^, impacting on future neuro-behavioural outcomes of those children affected. Unfortunately data on consanguinity are classified as highly sensitive information so were not available for use in this analysis.

There are also some limitations in the measures used. Loss of participants is minimised by the use of routinely collected, linked data. However, it remains possible that there was some loss of participants due to unsuccessful data linkage. For example, children not attending school were not captured. Rates of absence in state-funded primary schools in Bradford (4.4% overall rate of absence) were slightly above the figures for England as a whole (4%) so it remains possible that outcomes for the most vulnerable children were not considered^44^. Likewise, it is well documented that CMD is underdiagnosed in primary care^45,46^, including during the perinatal period^23^. Previously published analyses of the BiB cohort have also highlighted ethnic differences in detection of CMD within primary care, with some evidence that women of Pakistani ethnicity may be less likely to seek help for mental ill health^19^. The measure of CMD also does not capture the severity or chronicity of illness, which likely moderates the impact of antenatal CMD on offspring neuro-behavioural outcomes^6^.

## Conclusions

This study has highlighted the potential additional burden of exposure to common mental disorders in those children already affected by gestational diabetes during pregnancy. Therefore addressing inequalities in child development, whether in research, clinical practice, education or public policy, is likely to require a holistic approach to optimising maternal physical and mental health.

### Supplementary Information

Supplementary Information.

## Data Availability

Information about access to data and collaborations available at: https://borninbradford.nhs.uk/research/how-to-access-data/.
