# Supplementary material for: Child educational progress in Born in Bradford pregnancies affected by gestational diabetes and also exposed to maternal common mental disorders
Source: Sci Rep. 2023 Oct 21;13:17991. doi: 10.1038/s41598-023-44619-0 (PMC10590408; doi:10.1038/s41598-023-44619-0)
Supplement: Supplementary file 1 — Supplementary Information. [file 41598_2023_44619_MOESM1_ESM.pdf]

# Child educational progress in Born in Bradford pregnancies affected by gestational diabetes and also exposed to maternal common mental disorders

Claire A Wilson[1,2]*, Gillian Santorelli[3], Louise M Howard[1,2], Khalida Ismail[1,2], Rebecca M Reynolds[4], Emily Simonoff[1,2]

[1]Institute of Psychiatry, Psychology and Neuroscience, King's College London

[2]South London and Maudsley NHS Foundation Trust, UK

[3]Born in Bradford, Bradford Teaching Hospitals NHS Foundation Trust, Bradford, UK

[4]Centre for Cardiovascular Science, University of Edinburgh, UK

*Corresponding author address: PO31 King's College London, De Crespigny Park, London, SE5 8AF; e-mail: claire.1.wilson@kcl.ac.uk; ORCID ID: 0000-0003-2169-5115; tel: 07729 324100

Supplementary material S1: indicators of common mental disorders (CMD) from primary care records

***Prescriptions***

agomelatine, alprazolam, alventa, alventa xl, angilol, ativan, bonilux, bonilux xl, buspirone hydrochloride, chloralbetaine, chloralhydrate, chloral mixture bp2000, cipralex, cipramil, circadin, citalopram, clomipramine, clomipramine hydrochloride, clonezapam, depefex, depefex xl, diazepam, dosulepin, dosulepin hydrochloride, edronax, efexor, efexor xl, escitalopram, feprapax, fluoxetine, flurazepam, fluvoxamine, fluvoxamine maleate, foraven, foraven xl, gamanil, imipramine, imipramine hydrochloride, isocarboxazid, lofepramine, lomont, loprazolam, lorazepam, lormetazepam, lustral, manerix, marplan, melatonin, meprobamate, mianserin, mianserin hydrochloride, mirtazapine, moclobemide, molipaxin, nardil, nitrazepam, optimax, oxactin, oxazepam, parnate, paroxetine, phenelzine, politid, politid xl, propranolol, propranolol hydrochloride, prothiaden, prozac, ranfaxine, ranfaxine xl, reboxetine, seroxat, sertraline, sonata, stilnoct, surmontil, syprol, temazepam, tifaxin, tifaxin xl, tranylcypromine, trazodone, trazodone hydrochloride, trimipramine, tryptophan, valdoxan, venaxx, venaxx xl, venlafaxine, venlafaxine m/r, vensir, vensir xl, welldorm, winfex, winfex xl, zaleplon, zimovane, zispin, zispinsoltab, zolpidem, zolpidem tartrate, zopiclone, allegron, anafranil, anafranil sr, chlordiazepoxide, chlordiazepoxide hydrochloride, clomethiazole, cymbalta, doxepin, duloxetine, rivotril, sinepin, sodium oxybate, triptafen, xyrem, yentreve, amitriptyline, amitriptyline hydrochloride, nortriptyline, promethazine, promethazine hydrochloride

***Read codes***

*Depression:*
1B17., 1B19., 1B1U., 2257., 62T1., E112., E1120, E1121, E1122, E1123, E1125, E1126, E112z, E113., E1130, E1131, E1132, E1135, E1136, E1137, E113z, E118., E11y2, E11z0, E11z1, E11zz, E204., E210., E211., E2110, E2112, E2B.., E2B0., E2B1., Eu320, Eu321, Eu322, Eu324, Eu325, Eu326, Eu327, Eu32B, Eu32y, Eu32z, Eu330, Eu331, Eu33y, Eu33z, Eu34., Eu340, Eu34y, Eu34z, Eu3y., Eu3y1, Eu3yy, Eu3z., Eu53., Eu530, X00SO, X00SR, X00SS, X00SU, X00TX, X40Dl, X40Dm, X760u, X7617, X761I, X761J, X761K, X761L, XE0re, XE0uv, XE1Xy, XE1Y0,
XE1Y1, XE1YC, XE1ZY, XE1Za, XE1Zb, XE1Zc, XE1Zd, XE1Zf, XE1Zg, XE1Zh, XE1Zi, XE1aY, XE1ae, XM0Ar, XM0CR, XM1GC, XSEGJ, XSGok, XSGol, XSGom, Xa02E, Xa0wV, Xa110, Xa17z, Xa1eL, Xa9E0, Xa9J0, Xa9K0, XaCHr, XaCHs, XaCIs, XaCIt, XaCIu, XaImU, XaJWh, XaKUk, XaPKm, XaPOv, XaX0C, XaY2C, XaAyL, XaB5v, XaB95, XaB9J

*Anxiety:*
1B13., 1B1V., 2258., 225J., E0300, E0310, E200., E2000, E2001, E2002, E2004, E2005, E200z, E201., E2010, E2011, E2012, E2013, E2014, E2015, E2016, E2017, E2018, E201A, E201B, E201C, E201z, E202., E2020, E2021, E2022, E2023, E2024, E2025, E2026, E2027, E2028, E2029, E202A, E202B, E202C, E202D, E202E, E202z, E203., E2030, E2031, E203z, E205., E207., E20y., E20y0, E20y1, E20y2, E20y3, E20yz, E20z., E28.., E280., E281., E282.,
E283., E2830, E2831, E283z, E284., E28z., Eu40., Eu400, Eu401, Eu402, Eu40y, Eu40z, Eu41., Eu410, Eu411, Eu41y, Eu41z, Eu42., Eu420, Eu421, Eu422, Eu42y, Eu42z, Eu515, Eu51y, Eu51z, Ub1T9, X00Sc, X00Sf, X761N, XE0rb, XE1Y7, XE1YA, XE1Ym, XE1Yn, XE1Zj, XE1aW, XE1bo, XM1MZ, Xa0XG, Xa0XH, Xa0XI, Xa0XJ, Xa0XK, Xa0XM,

Xa0XN, Xa0XO, Xa0XP, Xa0XQ, Xa0XR, Xa0XX, Xa0XY, Xa0Xd, Xa18j, Xa18v, Xa19B, Xa3Xk, Xa3Ys, Xa7kB, XaEFB, XaP8d, XaX55, XaX56, XaX58

*Comorbid depression and anxiety:*
E2003, Eu412, Eu413, X00Sb

*CMD treatment or referral for CMD treatment:*
6655., 6659., 66590, 6779., 6G00., 8BK0., 8BM0., 8CQ.., 8CR7., 8F85., 8G..., 8G1.., 8G10., 8G100, 8G11., 8G12., 8G120, 8G121, 8G2.., 8G21., 8G2Z., 8G4.., 8G43., 8G4Z., 8G5.., 8G51., 8G5Z., 8G6.., 8G6Z., 8G7.., 8G7Z., 8G9.., 8G91., 8G9Z., 8HlB., 8HVO., 8H23., 8H230, 8H34., 8H38., 8H49., 8H7A., 8H7B., 8H7T., 8H7Z., 8HHp., 8HHq., 8HJ3., 8HK9., 8HkK., 8HM9., 9HZ.., 9N0T., 9N1M., 9N2B., 9N6h., 9NJ1., 9NJR., 9NJT., 9Ol.., Ub0qs, X71Ec, X71bp, X79sL, XE0iL, XE1Sa, XE1Sb, XSBbs, Xa8IB, Xa8IG, Xa8IJ, Xa8IP, Xa8IR, Xa8If, Xa8Ig, Xa8Ih, Xa8Ii, Xa8Ij, Xa8Ik, Xa8Is, Xa8It, Xa8Iu, Xa8Ix, Xa8J0, XaA8Z, XaA8c, XaA8d, XaA8u, XaA8v, XaA9W, XaA9g, XaABP, XaABQ, XaAKy, XaAMj, XaAMz, XaAOd, XaAOe, XaAOf, XaAOg, XaAOh, XaAQi, XaAQo, XaAS4, XaAU5, XaAUA, XaAXe, XaAZI, XaAbC, XaAbH, XaAdM, XaAel, XaAem, XaAen, XaAfJ, XaAh4, XaAiE, XaAiI, XaAkB, XaAkI, XaAkU, XaAnb, XaBHK, XaBIg, XaBJb, XaBJc, XaBT1, XaBTD, XaBtN, XaBvV, XaBvW, XaBvX, XaCFD, XaECG, XaEVq, XaI8j, XaINQ, XaINy, XaIOf, XaIOg, XaIOh, XaIOi, XaIOj, XaIOk, XaIOl, XaIOn, XaIOp, XaIOq, XaIOs, XaIOu, XaIOv, XaIOy, XaIOz, XaIP0, XaIP1, XaIP2, XaIP3, XaIPw, XaISp, XaISv, XaISw, XaISy, XaIT1, XaIT2, XaIT3, XaIT4, XaIT5, XaIT6, XaIT7, XaIT8, XaITA, XaITG, XaITH, XaITI, XaIUv, XaIUx, XaIUy, XaIUz, XaIV0, XaIV1, XaIV2, XaIV3, XaIV4, XaIV5, XaIV6, XaIW3, XaIW4, XaIW5, XaIW6, XaIWD, XaIWM, XaIWN, XaIWR, XaIWS, XaIWT, XaIWU, XaIWV, XaIWW, XaIWX, XaIWY, XaIWZ, XaIWa, XaIWb, XaIWx, XaIWy, XaIWz, XaIX0, XaIXS, XaIXT, XaIXU, XaIXV, XaIXW, XaIXX, XaIXY, XaIXZ, XaIXa, XaIXb, XaIXh, XaIXi, XaIXk, XaIXl, XaIXm, XaIXn, XaIXo, XaIXp, XaIXq, XaIXs, XaIXt, XaIXu, XaIYN, XaIkd, XaIkg, XaIku, XaIm4, XaIpA, XaItc, XaItx, XaIuR, XaIvk, XaIvp, XaIvq, XaIyU, XaJ4V, XaJ4w, XaJ4x, XaJOA, XaJON, XaJPu, XaJPz, XaJQ1, XaJQD, XaJQE, XaJQF, XaJQG, XaJQH, XaJQI, XaJQJ, XaJQR, XaJQS, XaJQT, XaJQU, XaJQV, XaJQW, XaJQX, XaJQY, XaJQZ, XaJRr, XaJWg, XaJr3, XaK1f, XaK5q, XaK5r, XaK6K, XaK70, XaK71, XaKAx,
XaKEz, XaKGq, XaKbb, XaL03, XaL0o, XaL0p, XaL0q, XaL0r, XaL0s, XaL0t, XaL0u, XaL0v, XaL0w, XaL2L, XaLBl, XaLCP, XaLCQ, XaLFL, XaLFk, XaLNF, XaLQw, XaLnp, XaLnq, XaLnr, XaLst, XaLsu, XaLsv, XaM2K, XaM7s, XaMGz, XaMJ8, XaMhM, XaN3a, XaN4b, XaN4c, XaN4d, XaN4e, XaN4f, XaN4g, XaNPL, XaNTc, XaONq, XaOOT, XaObo, XaOxM, XaP6T, XaP7x, XaPRF, XaPTT, XaPTU, XaPlZ, XaPvy, XaPvw, XaQBz, XaQC0, XaQWJ, XaQvz, XaR4n, XaR4s, XaR5D, XaWzW, XaX04, XaXEJ, XaXH8, XaXHm, XaXe3, XaXiH, XaXl2, XaY6o, XaY7i, XaYgS, XaZIW, XaZcf, ZV663, ZV673, ZV69., ZV690, ZV691, ZV692, ZV6D., ZV701, ZV702

*CMD-related follow-up:*
665.., 6654., 6658., 66580, 665A., 665A0, 665Z., 8A2.., 8A21., 8A2Z., 9H90., 9H91., 9H92., 9HA0., 9Ov.., 9Ov0., 9Ov1., 9Ov2., 9Ov3., 9Ov4., X74WN, XaJuG, XaJuK, XaJuT, XaJuV, XaJuW, XaK6d, XaK6e, XaK6f, XaK9p, XaKAK, XaLIb, XaMGL, XaMGN, XaMGO, XaMGP, XaMGQ, XaMGR, XaR9y, XaZ2p

*CMD History:*
146.., 1465., 1466., 1467., 146A., 146G., 146Z., 9HA1., Eu334, Xa41K, XaJWi, XaLG., ZV111

### *Timing of preconception CMD*

Women and all of their pregnancies were coded as affected by preconception CMD if there was attached to that woman any prescription or Read code for depression, anxiety, comorbid depression and anxiety, treatment of, follow up for or history of anxiety or depression dated prior to the date of conception of the first pregnancy; the first pregnancy for each woman may or may not have been a BiB pregnancy.
380 of 13,539 pregnancies in the sample had a missing date of conception due to unknown gestational age or date of delivery. In women with a pregnancy with a missing date of conception whose first pregnancy was a BiB pregnancy, if the date attached to the Read code or prescription was prior to 1 July 2006 (the earliest possible date of conception based on first recruitment into the study), these women and their pregnancies were recorded as affected by preconception CMD. Otherwise the woman was recorded as missing information about preconception CMD (it would seem erroneous for such women to take the default of no preconception CMD which was used for women in which no Read or prescription codes were recorded).

Women with recorded parity exceeding zero (i.e. multiparity) or missing parity in their first BiB pregnancy (i.e. births occurring prior to entry into BiB with unknown date of conception) and with a Read or prescription code, would also have had to be coded as missing, i.e. they may have had preconception CMD, but the date of conception of their first birth is not known. However, extraction of data from maternity records pertaining to pre-BiB pregnancies provided years of pregnancies prior to the BiB study for 5611 women. If the date of the primary care code preceded the earliest pregnancy date for that mother (only year was available so the date of pregnancy was elected to be the earliest possible at 1 January), she was coded as affected by preconception CMD. For multiparous women without this information available, she was recorded as missing information about preconception CMD following the same rationale as the paragraph above.

### *Timing of antenatal CMD*

Women were coded as experiencing antenatal CMD if there was a prescription or Read code for CMD in the mother’s records between the estimated date of conception and date of delivery.

Supplementary material S2: Complete case analysis of associations between antenatal CMD and EYFSP results in children exposed to GDM (N=632 children)

**Complete case analysis of associations between antenatal CMD and EYFSP results in children exposed to GDM (N=632 children)**

| | Failure to attain good level of development (GLD) on EYFSP | | | |
|---|---|---|---|---|
| **Unadjusted** | **%** | **RR** | **(95% CI)** | **p** |
| *Maternal indicator of antenatal CMD* Reference category= no indicator | 37.0 | | | |
| Antenatal CMD indicator | 45.3 | 1.18 | (0.87, 1.60) | 0.281 |
| **Adjusted*** | **%** | **RR** | **(95% CI)** | **p** |
| *Maternal indicator of antenatal CMD* Reference category= no indicator | 37.0 | | | |
| Antenatal CMD indicator | 45.3 | 1.19 | (0.89, 1.61) | 0.241 |

*Models using Poisson regression within a generalised estimating equation framework with robust standard errors *adjusted for maternal age, maternal education, multiple pregnancy, tobacco smoking in pregnancy, obstetric complications and maternal preconception CMD.*

Supplementary material S3: Associations between antenatal CMD and EYFSP sub-domain results in children exposed to GDM using imputed data and stratified by maternal ethnicity

**Associations between antenatal CMD and EYFSP sub-domain results in children exposed to GDM using imputed data and stratified by maternal ethnicity**

| | **Pakistani (N= 553 children)** | | | | **White British (N= 212 children)** | | | |
|---|---|---|---|---|---|---|---|---|
| | **Failure to attain good level of development (GLD) in communication and language** | | | | **Failure to attain good level of development (GLD) in communication and language** | | | |
| **Unadjusted** | **%** | **RR** | **(95% CI)** | **p** | **%** | **RR** | **(95% CI)** | **p** |
| *Maternal indicator of antenatal CMD* | | | | | | | | |
| Reference category= no indicator | 24.6 | | | | 19.0 | | | |
| Antenatal CMD indicator | 30.2 | 1.23 | (0.79, 1.92) | 0.350 | 13.9 | 0.76 | (0.22, 2.60) | 0.660 |
| **Adjusted*** | **%** | **RR** | **(95% CI)** | **p** | **%** | **RR** | **(95% CI)** | **p** |
| *Maternal indicator of antenatal CMD* | | | | | | | | |
| Reference category= no indicator | 24.6 | | | | 19.0 | | | |
| Antenatal CMD indicator | 30.2 | 1.24 | (0.80, 1.92) | 0.331 | 13.9 | 0.70 | (0.18, 2.71) | 0.602 |
| | **Failure to attain good level of development (GLD) in literacy** | | | | **Failure to attain good level of development (GLD) in literacy** | | | |
| **Unadjusted** | **%** | **RR** | **(95% CI)** | **p** | **%** | **RR** | **(95% CI)** | **p** |
| *Maternal indicator of antenatal CMD* | | | | | | | | |
| Reference category= no indicator | 37.0 | | | | 32.3 | | | |
| Antenatal CMD indicator | 50.5 | 1.36 | (1.01, 1.82) | 0.043 | 25.6 | 0.80 | (0.34, 1.93) | 0.626 |
| **Adjusted*** | **%** | **RR** | **(95% CI)** | **p** | **%** | **RR** | **(95% CI)** | **p** |
| *Maternal indicator of antenatal CMD* | | | | | | | | |
| Reference category= no indicator | 37.0 | | | | 32.3 | | | |
| Antenatal CMD indicator | 50.5 | 1.33 | (0.99, 1.78) | 0.061 | 25.6 | 0.75 | (0.29, 1.89) | 0.536 |
| | **Failure to attain good level of development (GLD) in maths** | | | | **Failure to attain good level of development (GLD) in maths** | | | |
| **Unadjusted** | **%** | **RR** | **(95% CI)** | **p** | **%** | **RR** | **(95% CI)** | **p** |
| *Maternal indicator of antenatal CMD* | | | | | | | | |
| Reference category= no indicator | 33.3 | | | | 29.3 | | | |

| | | | | | | | | | | |
|---|---|---|---|---|---|---|---|---|---|---|
| Antenatal CMD indicator | 43.2 | 1.29 | (0.92, | 1.81) | 0.138 | 24.8 | 0.87 | (0.36, | 2.11) | 0.755 |
| **Adjusted*** | **%** | **RR** | **(95% CI)** | | **p** | **%** | **RR** | **(95% CI)** | | **p** |
| *Maternal indicator of antenatal CMD* Reference category= no indicator | 33.3 | | | | | 29.3 | | | | |
| Antenatal CMD indicator | 43.2 | 1.27 | (0.91, | 1.79) | 0.161 | 24.8 | 0.75 | (0.29, | 1.94) | 0.557 |
| | **Failure to attain good level of development (GLD) in physical development** | | | | | **Failure to attain good level of development (GLD) in physical development** | | | | |
| **Unadjusted** | **%** | **RR** | **(95% CI)** | | **p** | **%** | **RR** | **(95% CI)** | | **p** |
| *Maternal indicator of antenatal CMD* Reference category= no indicator | 20.8 | | | | | 16.6 | | | | |
| Antenatal CMD indicator | 23.8 | 1.13 | (0.68, | 1.89) | 0.629 | 13.2 | 0.71 | (0.19, | 2.58) | 0.601 |
| **Adjusted*** | **%** | **RR** | **(95% CI)** | | **p** | **%** | **RR** | **(95% CI)** | | **p** |
| *Maternal indicator of antenatal CMD* Reference category= no indicator | 20.8 | | | | | 16.6 | | | | |
| Antenatal CMD indicator | 23.8 | 1.11 | (0.66, | 1.87) | 0.684 | 13.2 | 0.72 | (0.22, | 2.36) | 0.586 |
| | **Failure to attain good level of development (GLD) in personal, social and emotional domain** | | | | | **Failure to attain good level of development (GLD) in personal, social and emotional domain** | | | | |
| **Unadjusted** | **%** | **RR** | **(95% CI)** | | **p** | **%** | **RR** | **(95% CI)** | | **p** |
| *Maternal indicator of antenatal CMD* Reference category= no indicator | 21.8 | | | | | 19.8 | | | | |
| Antenatal CMD indicator | 24.0 | 1.10 | (0.66, | 1.81) | 0.722 | 17.9 | 0.85 | (0.30, | 2.43) | 0.760 |
| **Adjusted*** | **%** | **RR** | **(95% CI)** | | **p** | **%** | **RR** | **(95% CI)** | | **p** |
| *Maternal indicator of antenatal CMD* Reference category= no indicator | 21.8 | | | | | 19.8 | | | | |
| Antenatal CMD indicator | 24.0 | 1.09 | (0.66, | 1.81) | 0.739 | 17.9 | 0.91 | (0.32, | 2.64) | 0.868 |

*Models using imputed data in a Poisson regression within a generalised estimating equation framework with robust standard errors *adjusted for maternal age, maternal education, multiple pregnancy, tobacco smoking in pregnancy, obstetric complications and maternal preconception CMD*
